# Hindsight Experience Replay Improves Reinforcement Learning for Control of a MIMO Musculoskeletal Model of the Human Arm

**DOI:** 10.1109/TNSRE.2021.3081056

**Published:** 2021-06-08

**Authors:** Douglas C. Crowder, Jessica Abreu, Robert F. Kirsch

**Affiliations:** Department of Biomedical Engineering, Case Western Reserve University, Cleveland, OH 44106 USA, and also with the Functional Electrical Stimulation Center, Louis Stokes Cleveland VA Medical Center, Cleveland, OH 44106 USA

**Keywords:** Reinforcement learning, hindsight experience replay, actor-critic, musculoskeletal model, functional electrical stimulation

## Abstract

High-level spinal cord injuries often result in paralysis of all four limbs, leading to decreased patient independence and quality of life. Coordinated functional electrical stimulation (FES) of paralyzed muscles can be used to restore some motor function in the upper extremity. To coordinate functional movements, FES controllers should be developed to exploit the complex characteristics of human movement and produce the intended movement kinematics and/or kinetics. Here, we demonstrate the ability of a controller trained using reinforcement learning to generate desired movements of a horizontal planar musculoskeletal model of the human arm with 2 degrees of freedom and 6 actuators.The controller is given information about the kinematics of the arm, but not the internal state of the actuators.In particular,we demonstrate that a technique called “hindsight experience replay” can improve controller performance while also decreasing controller training time.

## I. Introduction

People with high-level (C1-C4) spinal cord injuries (SCIs) often develop paralysis of all four limbs - a condition known as tetraplegia. Functional electrical stimulation (FES) can be used to activate some paralyzed muscles and restore motor function to people with paralysis. When combined with an appropriate controller to coordinate the muscle contractions over time, as well as a command source that people can use to convey their intended movements, FES can produce functional movements [1], [2]. The movements produced by FES can greatly increase independence and quality of life for people with paralysis [1].

Recently, an FES system was demonstrated that used a brain-computer interface (BCI) as a command source [2]. The FES system was capable of selectively stimulating muscles of the upper arm, causing flexion and extension of the wrist and elbow, as well as hand opening and closing. The FES system was controlled using a set of piecewise linear functions that mapped a percent change in a BCI-decoded joint “command vector” to a percent change in the equilibrium position of the arm. The piecewise linear functions were manually tuned. While the controller enabled some restoration of motion, manual tuning was time consuming, and the results of that study suggested that the pre-defined piecewise linear functions lacked robustness against changes in posture.

Here, we seek to design an FES controller that works across a wide repertoire of movements by using methods of training that are quick and automatic. This controller should take, as inputs, the goal state of the movement rather than the (possibly suboptimal) movement trajectory. Additionally, the controller should only rely on easily-observable states, such as joint angle kinematics. Preferably, the chosen controller training method should, in the future, also be able to take subjective user feedback (e.g. “I like this controller better than that controller”) into account [3].

Several controller architectures have been proposed to control upper-limb FES systems that can be trained semi-automatically including: neural networks combined with proportional-integral-derivative (PID) controllers [4]–[6], neurofuzzy controllers [7], system identification [8], and reinforcement learning controllers [3], [9]–[14]. While many of these algorithms were able to control musculoskeletal systems, we determined that controllers based on reinforcement learning [15] could likely be adapted to meet the design criteria that are listed above.

Reinforcement learning controllers have previously been used to control upper-limb FES systems in musculoskeletal models [3], [9]–[12], virtual robots with muscle-like actuators [13], and humans [14]. Several of these controllers have achieved excellent performance in the tasks they were assigned, such as joint angle posture matching. However, most previous work has focused on designing controllers for systems that do not include multiple joints, biarticular muscles, or redundant, independently-controlled muscles. Furthermore, most previous work tested controller performance on tasks that included only one target or a small number of targets. It is unclear if the performance of these controllers will generalize to multi-input, multi-output (MIMO) systems that use biarticular, redundant, independently-controlled muscles to acquire targets placed at arbitrary locations within a workspace.

Recently, a reinforcement learning controller for an FES system was demonstrated that used the actor-critic method to achieve excellent performance in the metrics of “percent successful reaches” and “time to target” [3], [9]. It was demonstrated on a horizontal planar arm model with 2 degrees of freedom (DOF) and 6 actuators. However, training the controller required a pretraining step that utilized a proportional-derivative controller, which increased training time and controller design complexity. Additionally, training the controller required “reward shaping,” which refers to rewarding the controller for behaving in a way that is intuitive to humans. Reward shaping can limit reinforcement learning in many cases [16], including when the intuitive behavior is not the optimal behavior.

The controller presented here includes an improvement to the actor-critic reinforcement learning technique called hind-sight experience replay (HER) that was previously developed for controlling robots [17]. The main advantage of HER over other actor-critic reinforcement learning algorithms is the fact that HER can be trained with automatically-calculated, sparse rewards. The rewards do not require any shaping, and, in fact, sparse rewards are known to improve the performance of HER [17]. In addition, the proposed technique is model-free, uses readily-collectible state information (joint kinematics), is sampled at practical rates (50 Hz), and can be trained with limited to no involvement from investigators. Here, we demonstrate the utility of this controller using a multi-input, multi-output computational model of the human arm operating in the horizontal plane.

## II. Methods

### A. Musculoskeletal Model

To demonstrate the ability of a reinforcement learning approach to learn to control a relevant, dynamic, nonlinear musculoskeletal system, we used an existing model of the human arm, as described previously [3], [6], [9]. Motion of the arm was constrained to the horizontal plane, as if moving on a frictionless virtual tabletop. The model consisted of 2 segments: a forearm and upper arm, articulated by 2 joints (shoulder and elbow). Each joint was modeled as a pin joint with 1 degree of freedom. The model included 6 muscles as actuators. Four actuators were monoarticular, and two actuators were biarticular, roughly approximating the biceps, brachialis, posterior deltoid, anterior deltoid, and 2 heads of the triceps (long and short). The actuators were represented by Hill muscle models. Simulations were performed using forward Euler approximation, with model states updated every 20 ms.

**Fig. 1.**
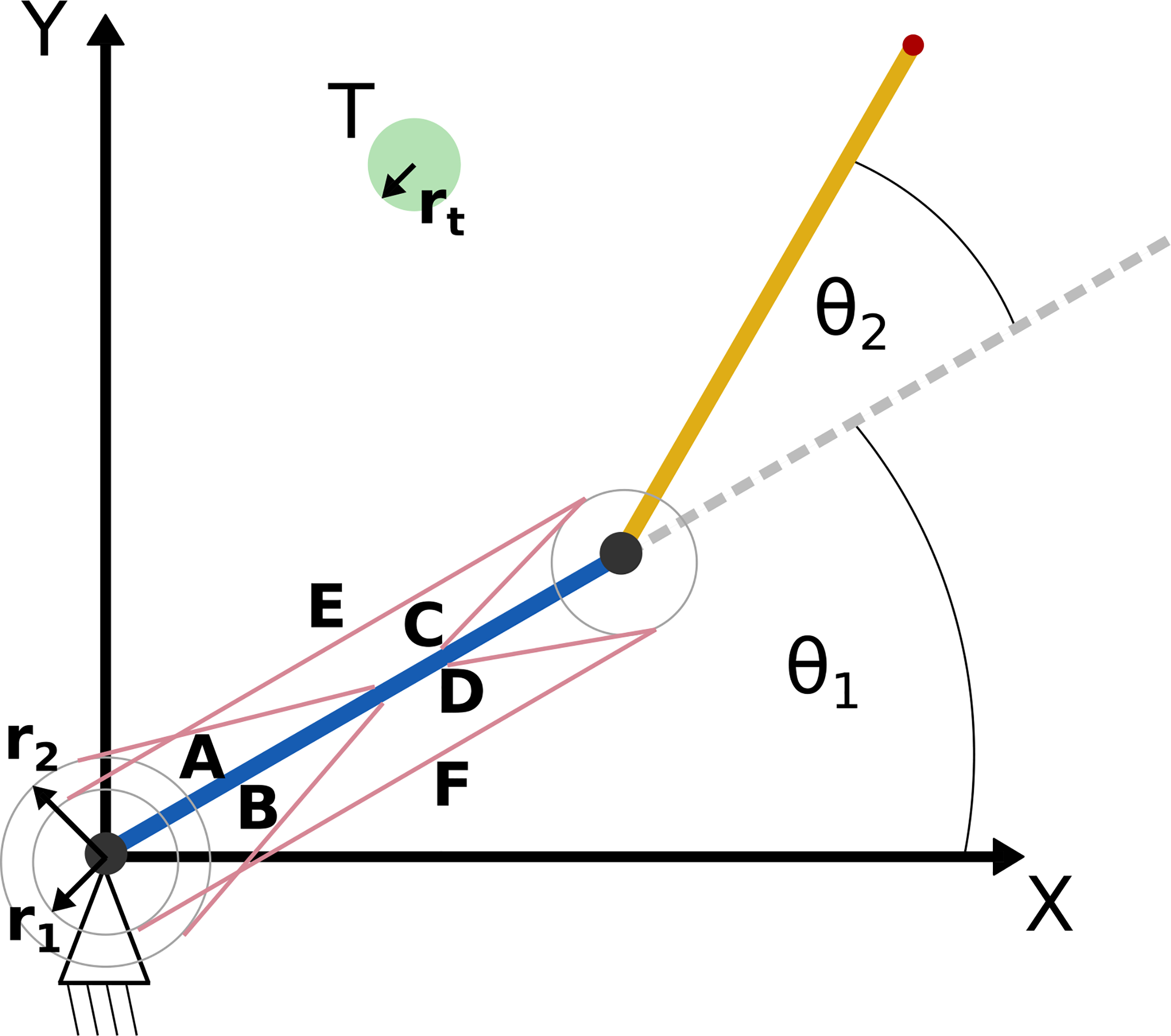
Illustration of the musculoskeletal arm model, as seen from above. The origin is at the shoulder. The positive y-axis is anterior, and the positive x-axis is anatomical right. The upper arm is represented by the blue line, and the lower arm is represented by the orange line. Actuators (in pink) are modeled after the following muscles: **(A)** anterior deltoid, **(B)** posterior deltoid, **(C)** brachialis, **(D)** triceps brachii (short head), **(E)** biceps brachii, **(F)** tricpes brachii (long head). Joint angles of the shoulder and elbow are denoted as θ_1_ and θ_2_, respectively. ***r***_**1**_ and ***r***_**2**_ denote the moment arms for the actuators.θ **T** denotes the target, which has radius ***r***_**t**._ Adapted from [3], [9].

To facilitate a direct comparison, we used the same parameters as [3], [9]. A diagram of the model is provided in Figure 1. Muscle parameters were derived from [18], [19], and limb segment parameters were derived from [20] for a person with a weight of 80 kg and a height of 177 cm.

### B. Task

The controller was tasked with moving the arm model to arbitrary locations within the arm’s horizontal plane workspace. To model real-world training paradigms, the arm started in the end state of the previous reach. The target region size and target spawning locations were chosen to match previous studies, which allowed for a more direct comparison [3], [9]. The target region for the reach was defined to be a circle with a radius of 7.5 cm. Targets were spawned within the joint angle range of [20°, 90°] for both the elbow and the shoulder. The distance between targets was 27±17 cm (mean ± standard deviation).

### C. Reinforcement Learning Controller

The upper-limb FES controller was trained using a reinforcement learning approach [15]. The controller observed the angular position and angular velocity of both the shoulder and the elbow. In addition, the controller observed the goal state (the final angular position) of the arm. The actions that the controller could take were the relative activations of each of the muscles over the range [0, 1]. Because the musculoskeletal model contained 6 actuators, the action space was a 6-dimensional vector at each time step. During each step, the reinforcement learning agent was given a reward of −0.1 to encourage exploration and quick movement to the target. When the endpoint of the arm reached the target region, the agent was given a reward of 1. Because the MIMO arm model had redundant muscles, constraints on learning were added to promote convergence. To this end, we penalized square root of the sum of the squared relative muscle activations with a reward of $-0.245*{\left(\sum_{i}a_{i}^{2}\right)}^{0.5}$, where *a*_*i*_ is the *i*^th^ element of the action (muscle activation) vector. The penalty on muscle activations encouraged the controller to find a control strategy that minimized the total muscle activation.

**Fig. 2.**
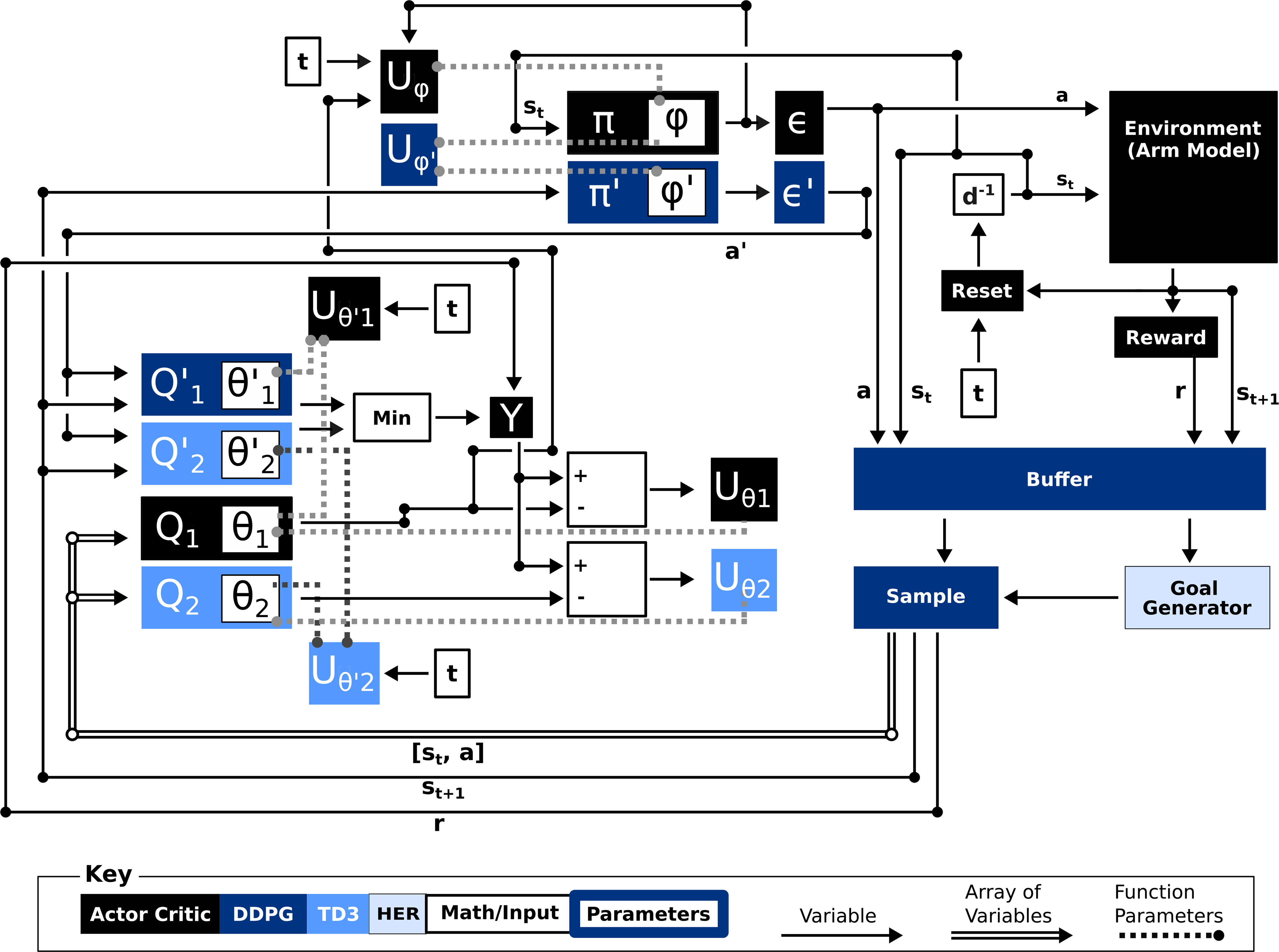
Overview of reinforcement learning algorithm. The vanilla actor-critic algorithm is a special case of DDPG, which is a special case of TD3, which is a special case of HER. Components of the controller added by each algorithm are colored, as described in the key. Unless specified otherwise, all variables are measured at time *t. s*_*i*_ is state at time *i*, a is the action, *r* is the reward, *d* ^*–*1^ is a delay of one time step, *Up* is a function acting on parameter set *p, θ* and *ϕ* are parameters, *π* is an actor, *Q* is the critic,*∊* is a noise function, and *Y* is a function computing the temporal-difference error.*F′*^´^ (note the apostrophe) denotes that function *F* is a target function. Compared to vanilla actor-critic methods, DDPG adds a sample buffer, a function to sample from the buffer, and “target” copies of the actor and critic networks that slow down parameter updates. Compared to DDPG, TD3 adds additional critic networks, which further slowdown parameter updates. Compared to TD3, HER adds a goal generator that allows the controller to learn from unsuccessful reaches by “pretending” that the goal of a reach was some position visited during the reach.

A brief summary of the reinforcement learning algorithm is provided, below, and also in Figure 2.

#### 1) Actor-Critic Methods:

Actor-critic reinforcement learning algorithms have previously been used in the literature to control musculoskeletal systems [3], [9]–[14]. Actor-critic algorithms use a function called an actor, *π*, that maps states (denoted as *s*) to actions (denoted as *a*), *s* → *a*. Actor-critic methods also use a function called a critic, *Q*, which maps [action, state] pairs to expected rewards (denoted as *E(r)*) *s* × *a* → *E(r)*. The output of the critic is used to estimate the reward that the agent will receive when observing state *s* and taking action *a*. The estimated reward is used to train the actor to maximize rewards. The critic is trained using temporal difference error [15]. Temporal difference learning causes the critic to update the predicted rewards based on observed rewards. Temporal difference methods assign rewards to actions while considering the temporal recency between actions and rewards. Here, as in previous works [3], [9]–[14], [21], [22], both the actor and the critic were neural networks.

#### 2) Deep Deterministic Policy Gradients:

Actor-critic methods have been observed to be unstable under several conditions, which motivated the development of Deep Deterministic Policy Gradients (DDPG) [21]. Firstly, quick updates to the critic parameters during training can cause unstable estimates of the expected reward. DDPG slows down parameter updates by introducing a second actor network *π′* and a second critic network, *Q′*. These so-called “target networks” store time-delayed versions of the actor and critic networks, and are used to stabilize critic parameter updates. Secondly, the statistics underlying how the critic estimates rewards assume independent observations. However, because actor-critic methods rely on observations that are continuous in time, temporally similar steps are not independent of one another. DDPG addresses this issue by introducing a buffer that stores past observations. The actor and critic are then trained using randomly-drawn observations from the buffer, rather than being trained on the current observations.

#### 3) Twin-Delayed DDPG:

Twin-Delayed DDPG (TD3) has been shown to improve reinforcement learning as compared to DDPG because TD3 stabilizes the learning gradients more than DDPG [22]. TD3 introduces the following innovations. Firstly, it uses two critic and two critic target networks. The minimum output of the two critic target networks is used as the training signal for the critic networks, which further decreases the variance in the learning gradients. Secondly, TD3 updates the critic networks more frequently than the actor networks and the target networks. This decreases how much the actor updates its weights based on poor estimates of action quality, thereby decreasing the probability that the actor network will produce divergent behavior.

#### 4) Hindsight Experience Replay:

Sparse reward strategies supply rewards only when a goal is met, which occurs infrequently for a controller taking random actions (hence, why they are called sparse rewards). In many regards, sparse reward strategies are the opposite of reward shaping strategies, which instead provide information about the quality of each action at each step. As discussed above, reward shaping strategies, if not carefully designed, can result in sub-optimal controller performance because, among other reasons, the subjective, human-intuitive assessment of the “best” action may not match the optimal action [16]. People with patient-specific motor disorders following SCIs may require non-standard movement paradigms to achieve goals, suggesting that reward shaping could be particularly detrimental when attempting to control paralyzed limbs. Unlike reward shaping strategies, sparse reward strategies do not provide much feedback on the quality of actions when goals are not met, which can result in slow learning. For this reason, we included Hindsight Experience Replay (HER) [17], which was designed to provide feedback about action quality when using sparse rewards.

HER is an algorithm that can be added to any off-policy reinforcement learning algorithm, such as DDPG and TD3, and that was developed for situations involving multiple goals, such as robotic reaching to arbitrary targets. When reinforcement learning agents attempt to learn in an environment with sparse rewards, unsuccessful reaches result in less information than successful reaches because the reinforcement learning algorithm does not know which action during the failed reach was wrong. In order to provide more information about the quality of actions made during failed reaches, HER samples arm reaching movements from a buffer, as in TD3 and DDPG, but instead of assigning the goal of the reach to be the original goal of the reach, HER considers the achieved (but incorrect) state of the reach to be the actual goal of the reach. Intuitively, the algorithm takes the approach: “I may not have wanted to reach to that location, but if I had wanted to reach to that location, I could have done so by taking the actions that I took.” By collecting information from multiple reaches, the actor learns to interpolate and reach arbitrary locations.

#### 5) Reinforcement Learning Hyperparameters:

As described above, HER retroactively reassigns the goal of the reach to be a state that was achieved during the reach. HER does this by breaking the reach into sub-reaches and assigning some state achieved during the sub-reach to be the goal of that sub-reach. The number of sub-reaches per reach that are added to the buffer is a hyperparameter (i.e., a parameter that affects the optimization process, rather than a parameter resulting from the optimization process) that needs to be specified. We specified the number of sub-reaches per reach to be 4, which was recommended in [17]. The method used to choose which state gets assigned as the goal of the sub-reach is also a hyperparameter that needs to be specified. We used the “future” method also recommended in [17], which means that the reassigned goal was chosen from the set of all achieved states observed after the sub-reach that occurred during the same reach.

During learning, reinforcement learning algorithms explore the action space by taking random actions during a set percentage of steps. We set the exploration parameter to be 0.3, meaning that 30% of actions were randomly drawn from a uniform distribution over the action space. During evaluation, the exploration parameter was set to 0 to better mimic real-world conditions.

For all conditions, the actors and critics were implemented using feedforward artificial neural networks with 2 layers and 64 units per layer.

Hyperparameters were tuned manually for the TD3-HER condition and held constant across all conditions.

#### 6) Software and Packages:

The musculoskeletal arm model was implemented in C [3], [6], [9]. Controller software was written in Python 3.6. Reinforcement learning was implemented using the Python package *stable-baselines* version 2.10.0 [23].

### D. Evaluation

For each condition, the controller training process was repeated 32 times. Each controller was trained for 100,000 20 ms timesteps (2,000 seconds or approximately 33 minutes, in total), which gave the HER-based controllers time to converge. Every 5 minutes of simulated training time, and at the end of training, evaluation was performed for 100 reaches. In order to assess learning over time and compare the present controllers with previous controllers, we used the following metrics (as used in previous work) [3], [9]:

#### 1) Fraction Success:

The controller was given 1 s to move the arm to the target region and remain in the target region, uninterrupted for 0.1 s. If the controller completed this task successfully, the trial was labeled as success. Otherwise, the trial was labeled a failure.

#### 2) Time to Target:

Time to target is called “time to dwell” elsewhere [3], [9]. It is defined as the time it takes for the controller to move the arm to the target region. Because the timeout was specified to be 1 second and the dwell time was defined to be 0.1 s, the maximum time to target was 0.9 seconds. Only successful reaches were included when calculating time to target.

#### 3) Fraction of Controllers Successfully Trained:

Not all controllers converged to parameters that allowed successful reaches to be made. Controllers were deemed to be “not successfully trained” if they did not acquire any targets during evaluation at the end of the training period.

In addition to these three metrics, the distributions of muscle activations and coactivations were plotted in order to determine the strategies used by trained controllers. When plotting the distributions, only controllers that acquired more than 90% of targets at the end of training were included. For the purposes of determining the degree of muscle coactivation, a muscle was considered active if its relative activation level was greater than 10%.

### E. Experiments

In our initial experiments, we tested if the controllers could control an arm when muscle forces were similar to muscle forces produced by able-bodied individuals. Because these controllers are being developed for people whose arms are stimulated via FES, we also tested if controllers could be trained to control an arm with muscle forces that are more likely for individuals with atrophied muscles due to paralysis, nominally equal to 50% of expected muscle forces for able-bodied individuals [3]. Then, to test the limits of the controllers, we decreased the target size progressively to targets of size [7.5, 5.0, 2.5, 1.25, 0.625] cm and asked the controller to move the arm to those target regions *without* retraining.

Throughout all experiments, we compared the performance of DDPG and TD3, with and without HER, to explore the improvements in performance due to each algorithm.

## III. Results

### A. Fraction of Controllers Successfully Trained

Table I summarizes the fraction of controllers that were successfully trained, meaning that they acquired at least one 7.5 cm target during the final 100-reach evaluation period. For each condition, 32 controllers were trained. Overall, 95% of controllers trained using HER could acquire targets, whereas only 52% of controllers trained without HER could acquire targets. For controllers trained using HER, 60/64 controllers trained using TD3 trained successfully, whereas 62/64 controllers trained using DDPG trained successfully. For controllers trained using HER, 62/64 trained successfully on a model of the arm using 100% of able-bodied muscle forces, and 60/64 trained successfully on a model of the arm using 50% of able-bodied muscle forces.

### B. Reinforcement Learning With 100% Muscle Forces

**Fig. 3.**
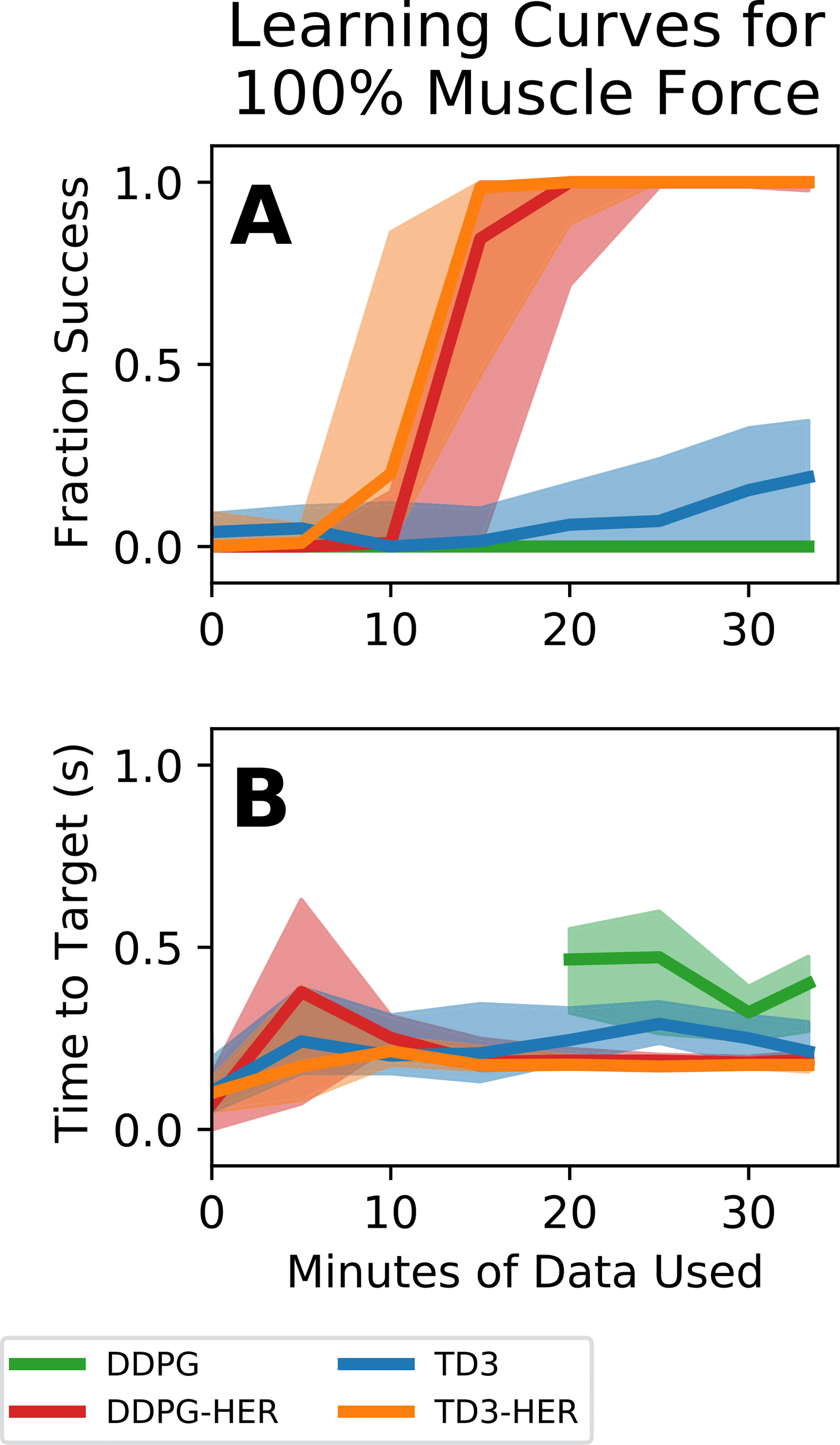
Training curves for arm with 100% muscle forces for different controller configurations. Bold lines represents median controller performance across all controllers of a given type. Shaded regions represent the performance of the 25^th^-75^th^ percentile controllers. **(A)** Fraction of reach trials successful as a function of minutes of simulated training data used. **(B)** Time to target as a function of minutes of simulated training data used. Controllers that used HER performed better than controllers that did not use HER. Controllers trained using DDPG without HER did not acquire any targets at early time points, meaning that time to target could not be calculated.

Figure 3 shows the learning curves for reinforcement learning algorithms trained on a model of an able-bodied arm. Algorithms that included HER (Figure 3a - red and orange curves) reached to targets successfully almost 100% of the time, whereas algorithms that did not include HER (Figure 3a - green and blue curves) reached to targets only about 10% of the time. As shown in Figure 3, controllers trained using HER (red and orange curves) plateaued in performance after approximately 20 minutes of training, whereas controllers that were not trained using HER (green and blue curves) did not complete training by the end of the 33 minute training period. Time to target typically increased from around 0 seconds at the beginning of training (because the controller only acquired targets that spawned on top of the endpoint of the arm) to around 0.3 seconds at the end of training (Figure 3b). Notably, controllers trained using DDPG without HER were unable to acquire any targets at the beginning of training, and times to target were higher than for all other conditions (Figure 3b, green curve).

### C. Reinforcement Learning With 50% Muscle Forces

**Fig. 4.**
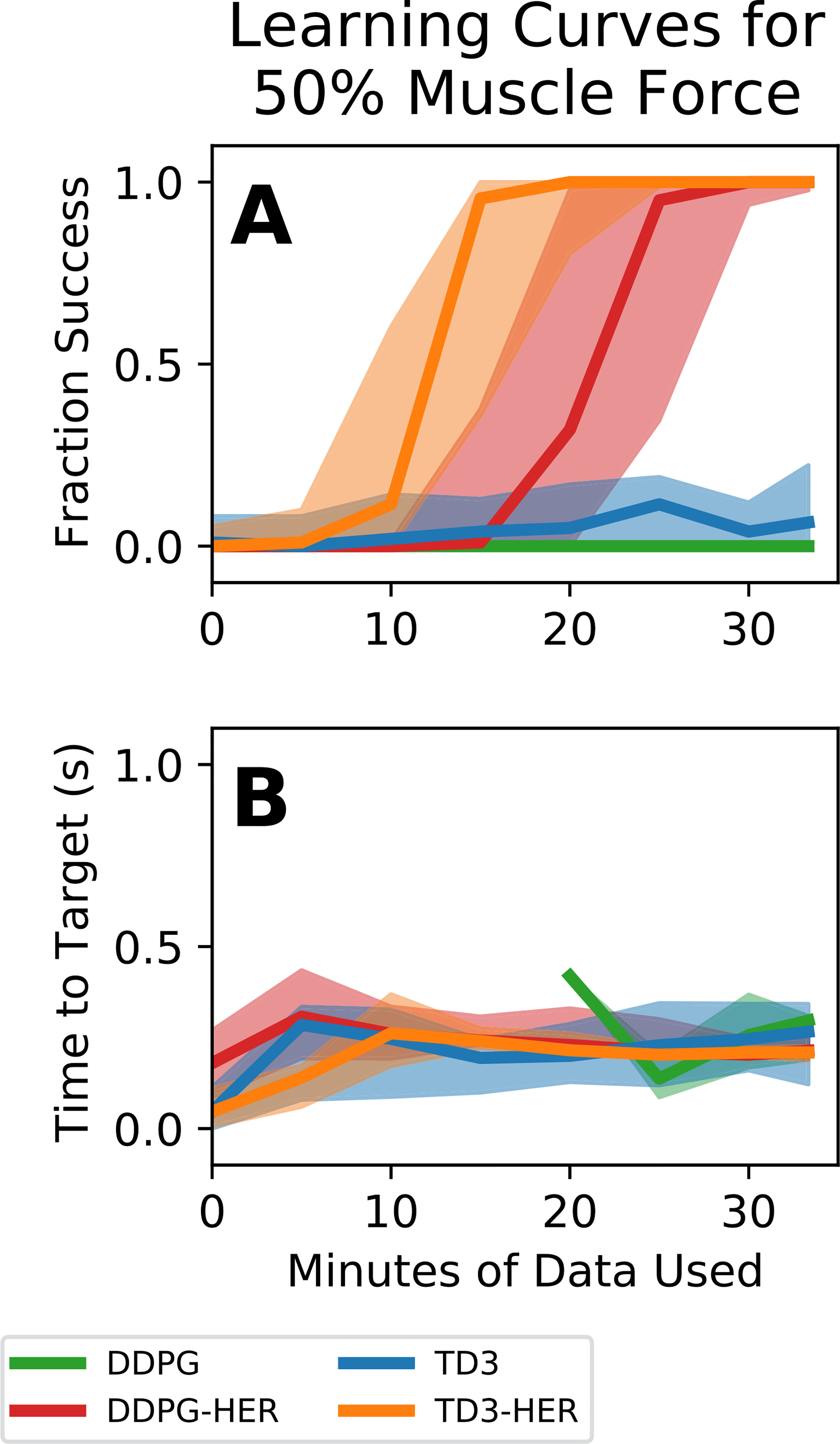
Training curves for arm with 50% muscle forces to simulate the reduced strengths of muscles following spinal cord injury. Bold lines represents median controller performance across all controllers of a given type. Shaded regions represent the performance of the 25^th^-75^th^ percentile controllers. **(A)** Fraction of reach trials successful as a function of minutes of simulated training data used. **(B)** Time to target as a function of minutes of simulated training data used. Controllers that used HER performed better than controllers that did not use HER. Controllers trained using DDPG without HER did not acquire any targets at early time points, meaning that time to target could not be calculated.

In order to model an FES-stimulated arm that has only 50% muscle strength (e.g., due to muscle atrophy or denervation following a spinal cord injury), we simulated an arm that could only produce 50% of the able-bodied muscle forces. Figure 4 shows the learning curves for reinforcement learning algorithms trained on a model of an arm with 50% muscle forces. Similar to the able-bodied condition, algorithms that included HER reached to targets successfully almost 100% of the time (Figure 4a - orange and red curves), whereas algorithms that did not include HER reached to targets only about 10% of the time (Figure 4a - blue and green curves). Controllers trained using HER plateaued in performance after approximately 25 minutes (TD3-HER) or 30 minutes (DDPG-HER) of training, whereas controllers that were not trained using HER did not complete training by the end of the 33 minute training period. in In the 100% muscle force condition, performance plateaued in 20 minutes, whereas for the 50% muscle force condition, performance plateaued in 25–30 minutes for controllers trained using HER. This suggests that decreasing muscle forces increased training time for controllers trained using HER. Like for the 100% muscle force condition, for the 50% muscle force condition, time to target typically increased from around 0 seconds at the beginning of training (because the controller only acquired targets that spawned on top of the endpoint of the arm) to around 0.3 seconds at the end of training. Notably, controllers trained using DDPG without HER were unable to acquire any targets at the beginning of training. (Figure 4b, green curve).

### D. Analysis of Controller Strategies

**Fig. 5.**
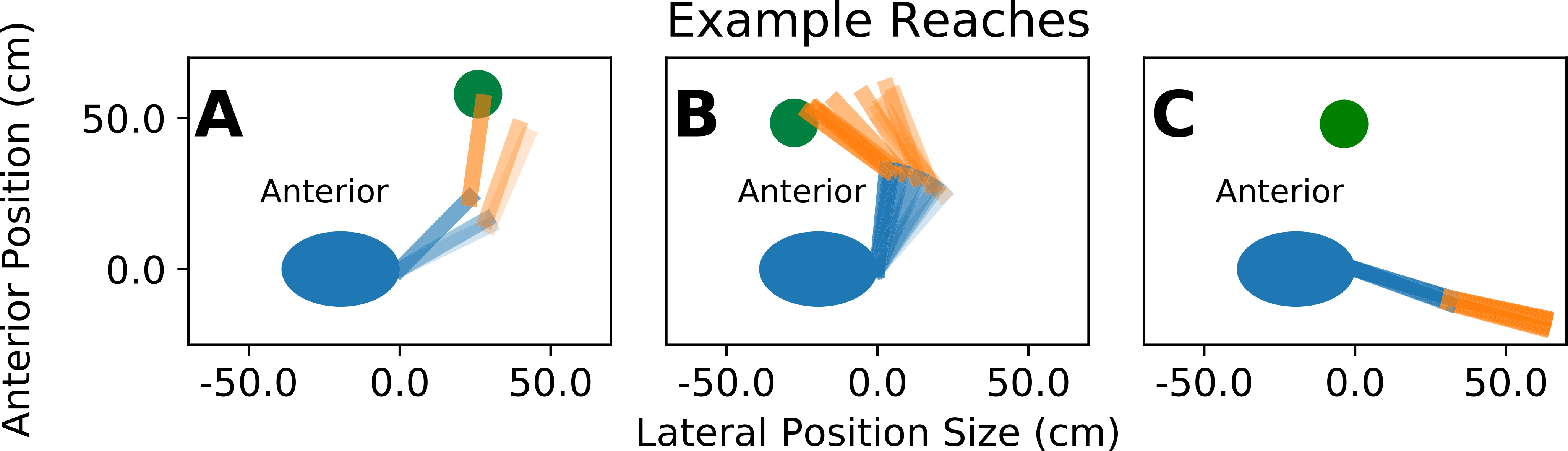
Examples of the three classes of arm reaches typically observed. The human body is represented by the blue ellipse. The target is shown in green. The upper arm is represented by a blue line, and the lower arm is represented by an orange line. Arm opacity increases as time progresses. **(A)** “Good” performance of a TD3 controller of an arm with 100% able-bodied muscle forces. Notice that the arm accelerates towards the target region, and decelerates as it nears the center of the target region. This control strategy was typical for controllers that were able to acquire 100% of targets. **(B)** “Borderline” performance of a DDPG-HER controller of an arm with 50% able-bodied muscle forces. Notice that the arm moves away from the target region in the beginning of the reach but then moves into the target region, though it does not reach the center of the target. This control strategy was typical for controllers that were able to acquire some, but not all, targets. **(C)** “Poor” performance of a TD3 controller of an arm with 100% able-bodied muscle forces. Notice that the arm remains mostly motionless near the joint angle limit. This control strategy was typical for controllers that were unable to acquire any targets.

Figure 5, shows example reach kinematics performed by controllers at the end of the training period. We observed 3 main controller strategies. The first strategy was characterized by rapid acceleration toward the target, followed by deceleration as the arm moved towards the center of the target (Figure 5a). This strategy was typical for controllers that acquired 100% of targets. The second strategy was characterized by unnecessary movements and target acquisition near the edges of the target region rather than at the center (Figure 5b). Frequently, controllers exhibiting this behavior only acquired some targets. The third controller strategy was characterized by small movements made near the limits of joint motion (Figure 5c). This strategy was typical for controllers that were unable to acquire any targets.

Supplementary Figure 1 shows the distribution of muscle activations commanded by successfully-trained DDPG-HER and TD3-HER controllers for the 100% muscle force condition. Similarly, Supplementary Figure 2 shows the distribution of muscle activations commanded by successfully trained DDPG-HER and TD3-HER controllers for the 50% muscle force condition. For both conditions and both controller configurations (DDPG-HER and TD3-HER), the controllers adopted similar strategies. 80% of the commanded muscle activation levels were either below 5% or above 95%.

Supplementary Figure 3 shows the distribution of the number of muscles that were coactivated for both the 100% and 50% muscle force conditions for all successfully-trained DDPG-HER and TD3-HER controllers. The distributions of coactivated muscles for all conditions and all controllers were similar. The distributions were left-skewed and had a single mode at 3 muscles activated. During 50% of time points, exactly 3 muscles were coactivated, and during another 38% of all time points, more than 3 muscles were coactivated, demon-strating that the solution found by the successfully-trained controllers relied on coactivation of many muscles.

### E. Testing 100% Muscle Force Controller on Smaller Targets

**Fig. 6.**
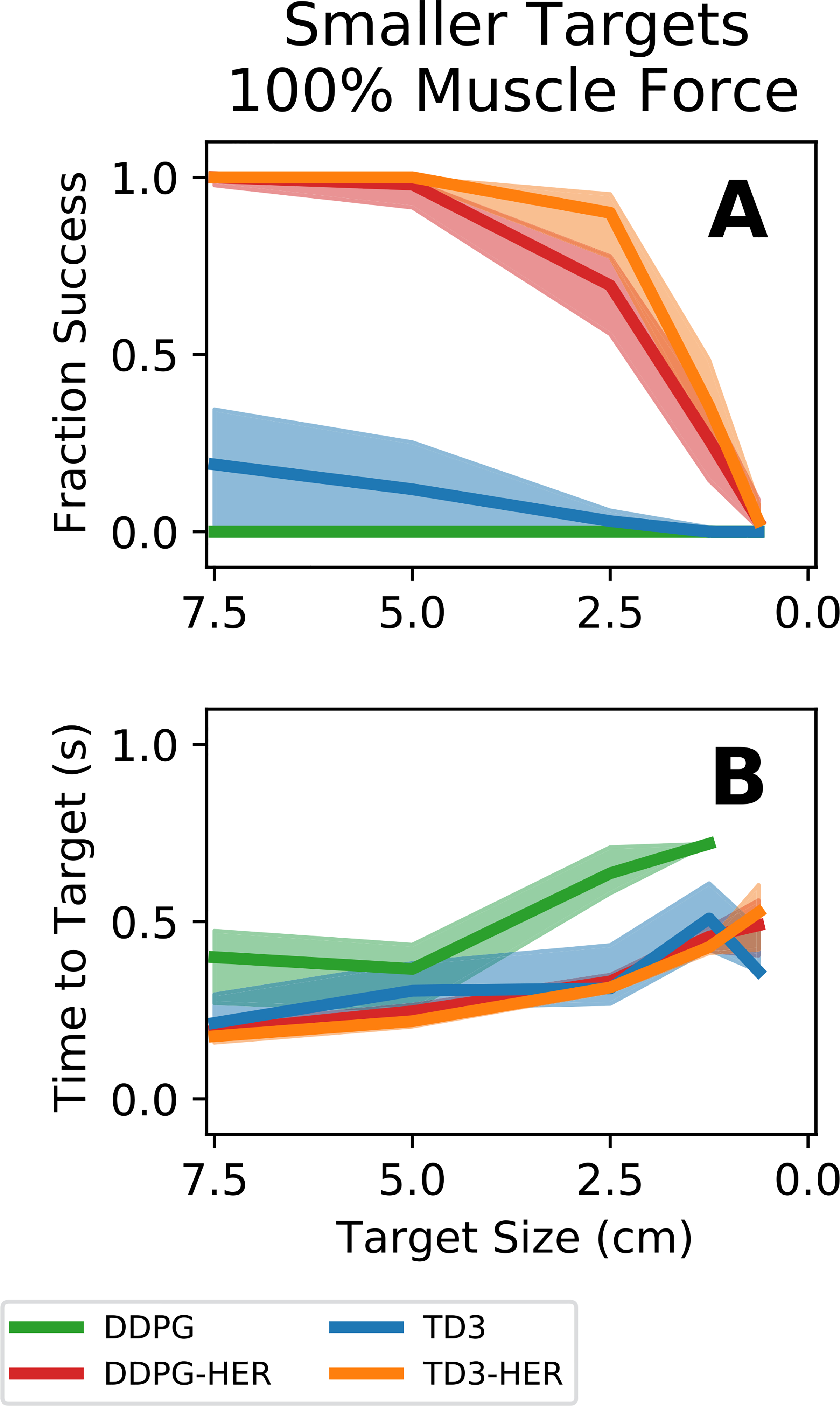
Controller performance on smaller targets with 100% muscle forces. Reinforcement learning agents were trained on 7.5 cm targets and then tested on smaller targets without retraining. Bold lines represent median performance across all controllers. Shaded regions represent the performance of the 25^th^-75^th^ percentile controllers. **(A)** Fraction successful as a function of target size. **(B)** Time to target as a function of target size. Targets above 5.0 cm in radius were acquired with little decrease in performance. Targets below 2.5 cm in radius were difficult for the controllers to acquire with no additional training. Controllers incorporating HER outperformed controllers that did not incorporate HER for all but the smallest targets.

To evaluate if a controller trained on 7.5 cm targets could also acquire smaller targets, we re-ran the simulated arm reaches on smaller targets in the range [7.5, 5.0, 2.5, 1.25, 0.625] cm. All controllers that used HER were able to acquire 5.0 cm targets (Figure 6 - orange and red lines). However, performance, as measured by fraction successful (Figure 6a) and time to target (Figure 6b), rapidly degraded for HER-based controllers when tested on targets smaller than 2.5 cm. Controllers that used HER (Figure 6 - orange and red lines) generalized to smaller targets better than controllers that did not use HER (Figure 6 - blue and green lines), although performance even for the HER-based controllers was rather poor for the smallest targets.

### F. Testing 50% Muscle Force Controller on Smaller Targets

**Fig. 7.**
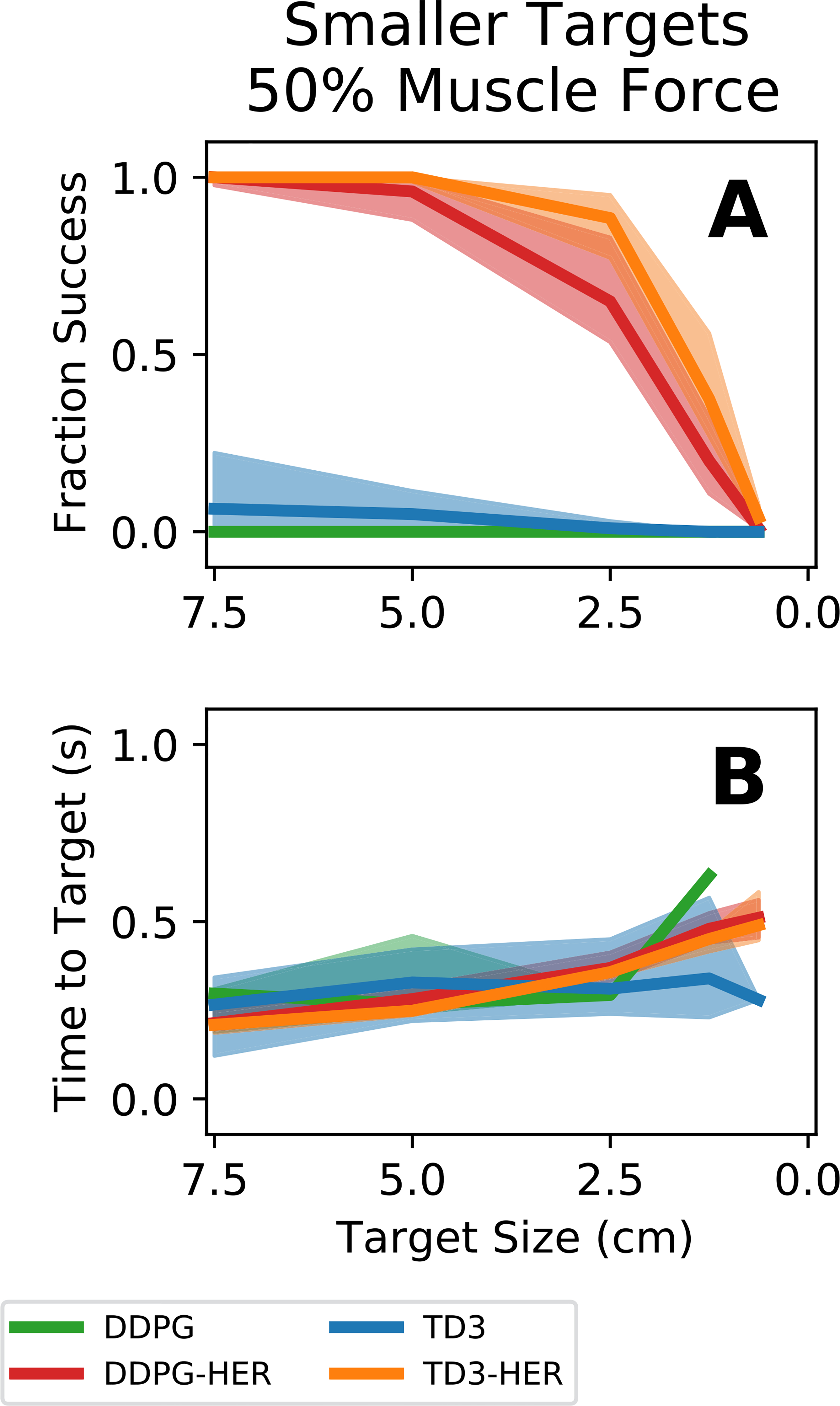
Controller performance on smaller targets with 50% muscle forces. Reinforcement learning agents were trained on 7.5 cm targets and then tested on smaller targets without retraining. Bold lines represent median performance across all controllers. Shaded regions represent the performance of the 25^th^-75^th^ percentile controllers. **(A)** Fraction successful as a function of target size. **(B)** Time to target as a function of target size. Targets above 5.0 cm in radius were acquired with little decrease in performance. Targets below 2.5 cm in radius were difficult for the controllers to acquire with no additional training. Controllers incorporating HER outperformed controllers that do not incorporate HER for all but the smallest targets.

Figure 7 summarizes the ability of the 50% muscle force controllers trained on 7.5 cm targets to generalize to smaller targets in the range [7.5, 5.0, 2.5, 1.25, 0.625] cm. All controllers that used HER were able to acquire 5.0 cm targets (Figure 7 - orange and red lines). However, performance, as measured by fraction successful (Figure 7a) and time to target (Figure 7b), rapidly degraded for HER-based controllers when tested on targets smaller than 2.5 cm. Controllers that used HER (Figure 7 - orange and red lines) generalized to smaller targets and weaker muscles better than controllers that did not use HER (Figure 6 - blue and green lines). Again, however, performance even for the HER-based controllers was rather poor for the smallest targets.

## IV. Discussion

### A. Controller Training Successes

In this study, controllers incorporating HER were trained successfully over 95% of the time. For the remaining 5% of unsuccessful controller training attempts (i.e., when the controllers were unable to reach any targets), a new controller could be trained quickly. The methods used here only allow one controller to be trained at a time. However, this is not a major concern since training a new controller takes only 20–25 minutes.

Example reaches shown in Figure 5 shed light on why controllers sometimes fail to train. After training was complete, we qualitatively examined the behavior of the controllers and observed the following. Controllers that were able to acquire nearly 100% of 7.5 cm targets accelerated the arm towards the target and then decelerated as the arm approached the center of the target (Figure 5a). Controllers that were able to acquire some targets frequently moved indirectly towards the target and acquired the target near the edge of the target region (Figure 5b). In a few cases, we provided these controllers with additional training time, and the controllers were able to learn to acquire nearly 100% of targets, suggesting that this behavior can be resolved by providing additional training time, or simply by retraining. Finally, controllers that failed to acquire any targets at the end of training tended to remain nearly motionless near the limits of motion (Figure 5c). These controllers typically output relative muscle stimulation values that were close to 0. Providing additional training time to controllers that did not produce movement did not improve results, suggesting that these controllers found local maximums in the reward function by minimizing muscle activity without acquiring any targets.

Taken together, these results suggest a mechanism for the success of HER-based algorithms in controlling FES-actuated MIMO human arms. HER provides feedback to the reinforcement learning algorithm, even when the rewards are sparse, which leads to faster learning. If the controller learns to move the arm to targets faster than it learns to minimize muscle activity, then learning occurs, and at least some targets can be acquired. However, if the controller learns to minimize muscle activity much faster than it learns to move the arm, the arm will remain mostly motionless.

### B. Comparison to Previous Results

The performances of the TD3-HER and DDPG-HER controllers compare favorably to the performances of reinforcement learning controllers used in recent work for controlling a 2D model of the human arm [3], [9]. Previous controllers were able to acquire about 90% of targets with a 2 second timeout. In this paper, algorithms incorporating HER acquired nearly 100% of targets with a 1 second timeout. In previous papers, the best time to target was approximately 1.4 seconds, to the best of our knowledge. Here, the time to target was approximately 0.3 seconds for controllers incorporating HER.

All HER algorithms presented here can learn using pure reinforcement learning, unlike previous reinforcement learning works using MIMO musculoskeletal systems, which required pretraining using data from a proportional-derivative feedback controller [3], [9]. Pretraining steps require more data, longer training times, and more investigator involvement in training. Additionally, pure reinforcement learning approaches may generalize better when systems include more complex links between degrees of freedom and actuators because reinforcement learning does not require manual tuning of the architecture (such as in cascading proportional-integral-derivative controllers and model-based controllers). Other reinforcement learning approaches for training FES controllers used a pretraining step involving a patient-specific model of the participant’s arm [14]. While pretraining with a patient-specific model could decrease training times further, the results of this study suggest that using DDPG-HER or TD3-HER algorithms can be used to efficiently train FES controllers in the absence of patient-specific models. This feature of DDPG-HER and TD3-HER controllers could be particularly useful for more complex biomechanical systems that have not been adequately modeled or for people who have anatomical anomalies that are not easy to represent in models.

Most of the parameters used in this paper matched those used by Jagodnik *et al.* [3], [9]. However, we chose to model decreases in muscle forces as uniform decreases in all muscle forces rather than choosing to decrease only flexor muscle forces, as chosen by Jagodnik, *et al.* because uniform decreases in muscle forces more closely match our observations of people with SCIs.

### C. Comparison to Able-Bodied Control

Controllers incorporating HER were able to reach to nearly 100% of targets, and time to target for these controllers plateaued at around 0.3 seconds for 7.5 cm targets - a time to target that is highly consistent with well-known speed-accuracy tradeoffs for human goal-oriented movements, such as Fitt’s Law [24]. Thus, HER-based controllers perform comparably to able-bodied humans for both the 100% muscle force and 50% muscle force conditions.

### D. Algorithm Choice

Although HER and TD3 incur additional computational costs during training compared to DDPG, the features responsible for this added computational load (i.e., the critic and target networks, as well as the goal reassignment function) are actually only necessary during controller training. Once training is complete, the cost of using each controller is the same because only the actor is used. Furthermore, training can typically be performed on a stand-alone machine, rather than the embedded system used for control. So, the choice of algorithm is not constrained by computational power. Thus, we can choose the algorithm based on anticipated performance alone. Even so, the training computational costs were quite modest - the most computationally-intensive algorithm, TD3-HER, only used about 500 MB of RAM and 20% of the computational power of a machine with a quad-core Intel Core i7–4810MQ CPU operating at 2.8 GHz. The entire training process, including simulation of 33 minutes of movement, took only 6 minutes. Thus, the duration of data collection, not computational power, was the limiting factor for the learning rate of the controllers.

In general, reinforcement learning algorithms (as applied to controlling arm movements) incorporating HER outperform algorithms that do not incorporate HER in terms of the fraction of successfully-trained controllers, the fraction of successful reaches, and the time to target. Thus, we recommend using algorithms incorporating HER for controlling FES-actuated arms. Choosing between TD3-HER and DDPG-HER is difficult, and the results suggest that either algorithm is suitable for training controllers of FES-actuated arms.

### E. Evaluation of the Controller Strategy

Successfully-trained TD3-HER and DDPG-HER controllers adopted similar strategies. As show in Supplementary Figures 1 and 2, TD3-HER and DDPG-HER controllers took an approach to muscle stimulation that approximated “all or nothing.” As shown in Supplementary Figure 3, DDPG-HER and TD3-HER controllers preferred to stimulate 3 of the 6 muscles most of the time and preferred to stimulate more than 3 muscle rather than less than 3 muscles. This strategy was nearly the same across all controllers and all muscle force conditions. To move a system with 2 mechanical degrees of freedom in the desired direction, at least 2 muscles should be activated simultaneously. Coactivation of 3 or more muscles might be used to accelerate the arm more quickly or to provide stiffness that can dampen oscillations.

### F. Real-World Deployment

The strategies used by the successfully-trained controllers resulted in smooth movements to the targets, with speeds comparable to human movement. Relative muscle activation levels tended to be less than 5% or more than 95%. This “all or nothing” approach to muscle activation could potentially induce fatigue, be uncomfortable to the participant, or even cause injury. For this reason, we recommend that, before controller training, each electrode should be profiled to set safe thresholds for stimulation, as is common practice [2]. In this study, the arm was allowed to move quickly in order to demonstrate the limits of training speed and performance. However, if slower movements are desired (for instance, because of safety concerns), explicit constraints can be placed on the controller by limiting muscle activation levels or penalizing speed in the reward function.

In previous works, a pretraining step was frequently used. When using HER, pretraining is not required, which could be beneficial for increasing the training speed and decreasing the involvement of health care practitioners.

The model used here made the implicit assumption that activation of muscles is selective and time-invariant. These assumptions are reasonable for highly-selective implanted electrodes, as used previously [2].

### G. Limitations

In order to understand how reinforcement learning can be used to train controllers for the human arm, we chose to use a simplified model of the human arm, as done previously [3], [9], that had fewer mechanical degrees of freedom and fewer actuators than a human arm [3], [9]–[14] and also that did not include gravity [3], [9]–[14], muscle fatigue [3], [9]–[13], or time-varying dynamics such as those caused by muscle spasticity [3], [9]–[13]. We made this decision specifically to evaluate the different reinforcement learning algorithms before applying them to more complex and realistic arm models. The model of the human arm used here is comparable to other musculoskeletal systems used to evaluate the performance of reinforcement learning controllers. Further studies are necessary to evaluate reinforcement learning controllers in a more complete model of the human arm.

The hyperparameters that we chose were based on previous work, and then manually-tuned. Choosing an optimal set of hyperparameters may improve learning further, and may help elucidate differences between TD3-HER and DDPG-HER algorithms.

## V. Conclusion

We have demonstrated that reinforcement learning algorithms incorporating HER improve control of a multi-input, multi-output musculoskeletal model of the human arm. Controllers trained using HER are more likely to learn to control the arm model within the specified training time, they learn more quickly, and they produce better control than reinforcement learning algorithms that do not use HER. Controllers trained using HER approach able-bodied performance for 7.5 cm targets in terms of the number of targets successfully acquired and the time required to acquire targets.

## Supplementary Material

supp1-3081056

## Figures and Tables

**TABLE I:** Fraction of Controllers Successfully Trained

Maximum Muscle Force (%)	DDPG	DDPG-HER	TD3	TD3-HER
50.0	0.22	0.94	0.78	0.94
100.0	0.34	1.00	0.75	0.94
