# Supplementary material for: Hindsight Experience Replay Improves Reinforcement Learning for Control of a MIMO Musculoskeletal Model of the Human Arm
Source: IEEE Trans Neural Syst Rehabil Eng. Author manuscript; Available in PMC 2021 Nov 30. (PMC8630802; doi:10.1109/TNSRE.2021.3081056)
Supplement: supp1-3081056 [file NIHMS1712878-supplement-supp1-3081056.pdf]

Supplementary Information: Hindsight Experience Replay Improves Reinforcement Learning for Control of a MIMO Musculoskeletal Model of the Human Arm

Douglas C. Crowder, Jessica Abreu, and Robert F. Kirsch

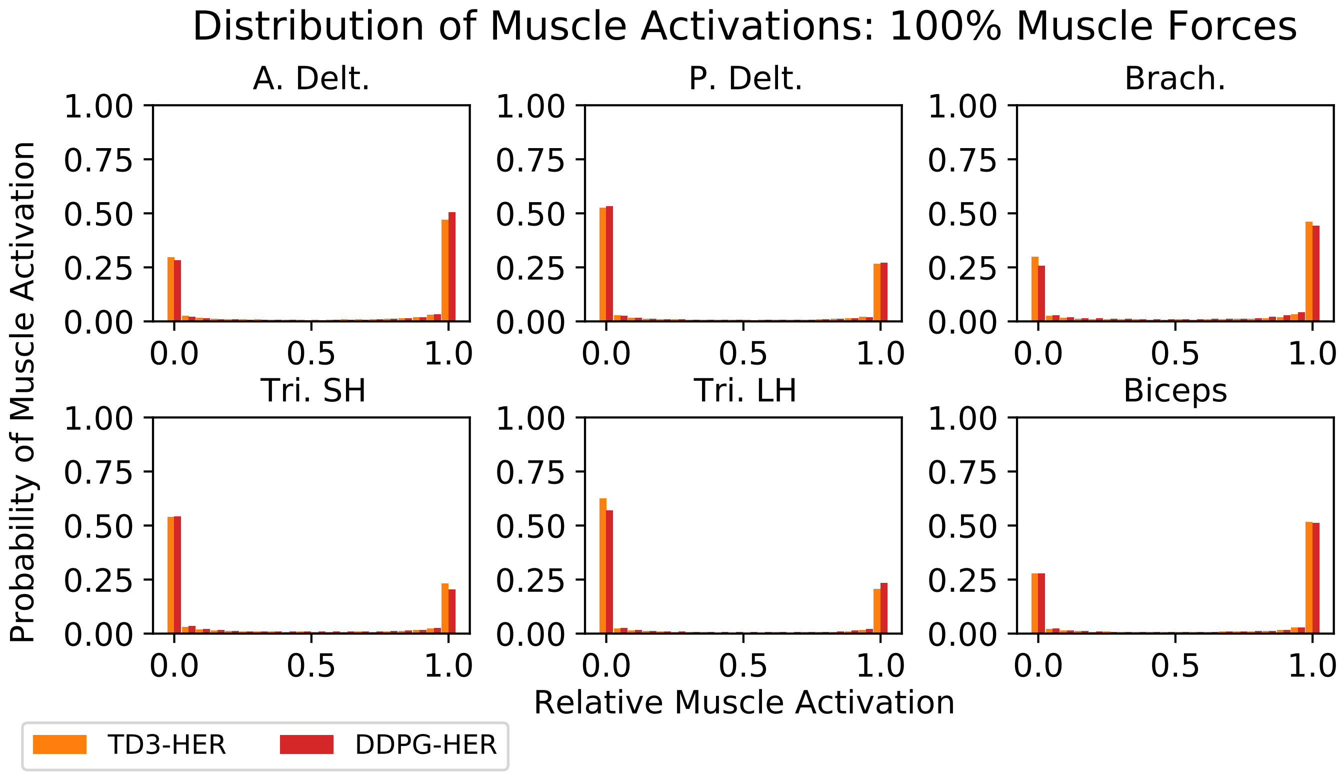

Supplementary Fig. 1. **Distribution of muscle activations for 100% muscle force condition.** DDPG-HER (red) and TD3-HER (orange) controllers were trained for 100,000 timesteps (approximately 33 minutes) and performance was evaluated. For controllers that acquired more than 90% of targets, muscle activation values commanded by the controller were collected. The distribution of muscle activations for all successfully-trained controllers has been plotted. The distributions of activations are nearly identical for the DDPG-HER and TD3-HER conditions. Muscles tend to be nearly 100% active or nearly 0% active.

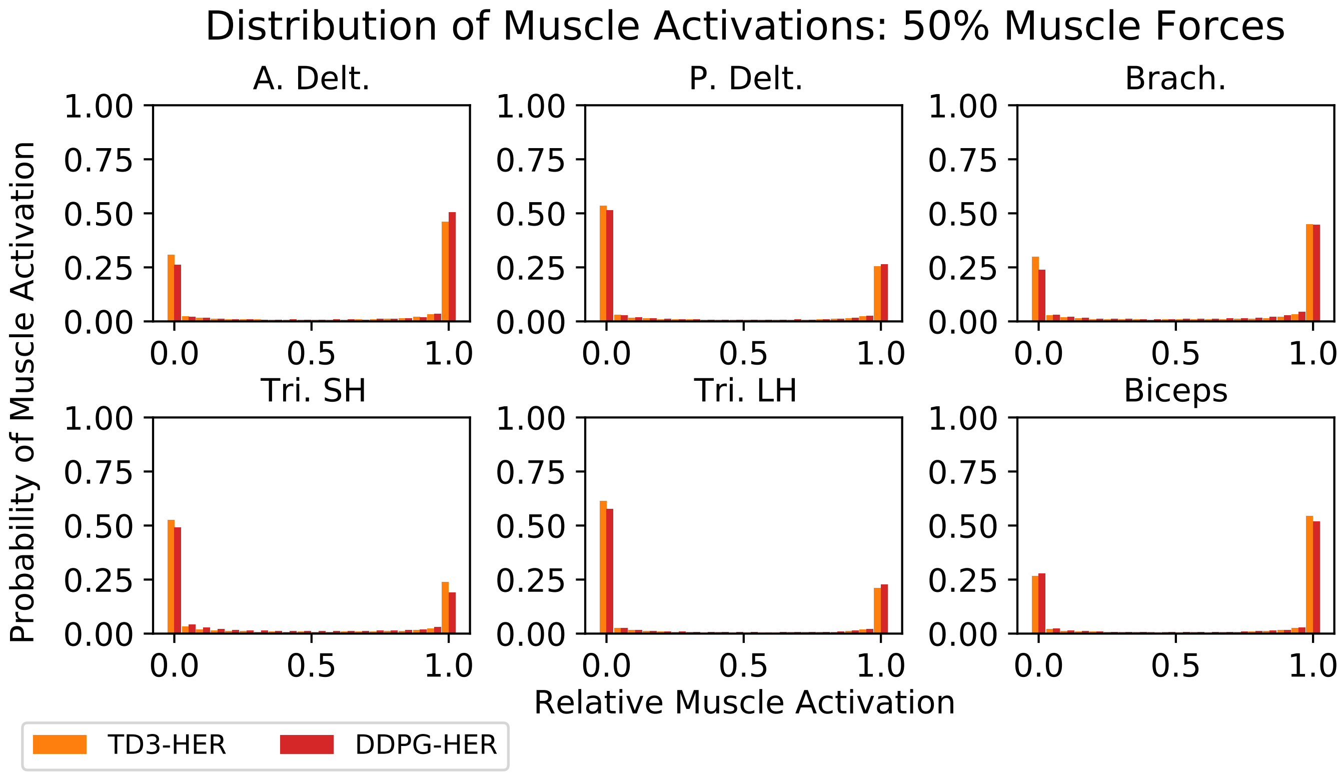

Supplementary Fig. 2. **Distribution of muscle activations for 50% muscle force condition.** DDPG-HER (red) and TD3-HER (orange) controllers were trained for 100,000 timesteps (approximately 33 minutes) and performance was evaluated. For controllers that acquired more than 90% of targets, muscle activation values commanded by the controller were collected. The distribution of muscle activations for all successfully-trained controllers has been plotted. The distributions of activations are nearly identical for the DDPG-HER and TD3-HER conditions. Muscles tend to be nearly 100% active or nearly 0% active.

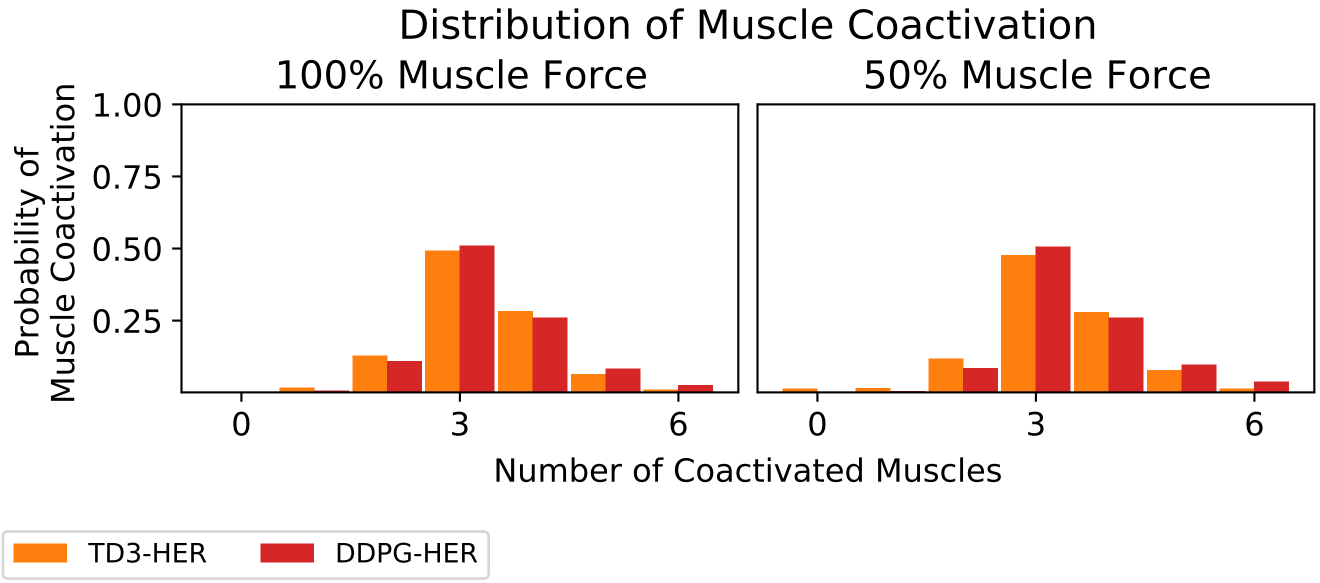

Supplementary Fig. 3. **Distribution of number of coactivated muscles.** DDPG-HER (red) and TD3-HER (orange) controllers were trained for 100,000 timesteps (approximately 33 minutes) and performance was evaluated. For controllers that acquired more than 90% of targets, muscle activation values commanded by the controller were collected. The distribution of the number of muscles activated more than 10% at any point in time has been plotted. The distributions of coactivations are similar for the DDPG-HER and TD3-HER conditions. 3 or more muscles tend to be coactivated for both the 100% muscle force and 50% muscle force conditions.
